# Use of Mobile Sensing Data for Longitudinal Monitoring and Prediction of Depression Severity: Systematic Review

**DOI:** 10.2196/57418

**Published:** 2025-08-21

**Authors:** Rebeka Amin, Simon Schreynemackers, Hannah Oppenheimer, Milica Petrovic, Ulrich Hegerl, Hanna Reich

**Affiliations:** 1 Research Centre of the German Foundation for Depression and Suicide Prevention Department for Psychiatry, Psychosomatics and Psychotherapy, University Hospital Goethe University Frankfurt Frankfurt am Main Germany; 2 German Foundation for Depression and Suicide Prevention Leipzig Germany; 3 Division of Mental Health and Substance Abuse Diakonhjemmet Hospital Oslo Norway; 4 Department of Psychology University of Oslo Oslo Norway; 5 Goethe Research Professorship Department for Psychiatry, Psychosomatics and Psychotherapy, University Hospital Goethe University Frankfurt Frankfurt am Main Germany

**Keywords:** depression, depressive disorder, smartphone, smartwatch, wearable device, sensor data, mobile sensing, digital phenotyping, monitoring, tracking, prediction methods, forecasting, algorithms, machine learning, systematic review

## Abstract

**Background:**

Depression is highly recurrent and heterogeneous. The unobtrusive, continuous collection of mobile sensing data via smartphones and wearable devices offers a promising approach to monitor and predict individual depression trajectories, distinguish illness states, and anticipate changes in symptom severity.

**Objective:**

This systematic review evaluates whether objective data from wearable devices and smartphones can (1) monitor and distinguish different states of depression, (2) predict changes in symptom severity, and (3) identify clinically relevant objective features for tracking and forecasting depression within diagnosed individuals.

**Methods:**

We searched PubMed and Web of Science databases for English-language studies (published 2012-2022) that used smartphone or wearable device data, included participants aged ≥14 years with a depression diagnosis, and collected continuous data for at least 12 weeks.

**Results:**

Out of 12,997 peer-reviewed articles, 9 original studies met the inclusion criteria, with sample sizes ranging from 45 to 2200 and durations of 12-52 weeks. Of the 9 studies, 3 used smartphone data, 1 used wearable device data, and 5 used both data types. Commonly collected variables were step count, distance moved, smartphone usage, call logs, sleep, heart rate, light exposure, and speech patterns. One study (11%) successfully differentiated between depressive states (worsening, relapse, or recovery). Six studies (67%) showed that mobile sensing data could predict depressive episodes or symptom severity. Four studies reported the predictive accuracy for depression using mobile sensing data from smartphones and wearable devices, ranging from 81% to 91%. Higher accuracy was achieved with personalized models or multimodal data.

**Conclusions:**

Real-time passive monitoring via wearable devices and smartphones holds promise for personalized self-management, but key gaps remain, such as a lack of longitudinal and long-term studies with data collection for 1 year or longer, studies with confirmatory parameters on an individual level, and studies with a strong correlation between parameters in individual patients to support clinical decision-making. Improvements in reporting standards are highly recommended to provide better-informed insights for clinicians. Throughout this process, there is a clear need to address various other issues, such as limited types of collected data, reliability, user adherence, and privacy concerns.

**Trial Registration:**

PROSPERO CRD42022355696; https://www.crd.york.ac.uk/PROSPERO/view/CRD42022355696

## Introduction

### Background

Depression (International Classification of Diseases, version 10 [ICD-10]: F32, F33) is a highly prevalent, usually recurrent or chronic, severe, and sometimes life-threatening disease [1]. The World Health Organization proclaimed depression as one of the leading causes of disease and disability in the world [2]. The duration of major depressive episodes has been found to vary widely, with median durations ranging from 3 to 6 months [3-6]. The Collaborative Depression Study reported that a primary episode usually recovers within 1 year, and the initial recurrence rate is 25%-40% after 2 years [4]. Broadly, individuals with depression might experience 5 to 9 episodes of depression in their lifetime, with each episode potentially presenting with worsened symptoms and having a progressively devastating impact on well-being [7]. In between the episodes, there are symptom-free intervals, during which well-managed strategies might prevent the risk of a worsening status [8,9]. If the period of remission and recovery could be guided by personalized self-management strategies, it would improve the experience and quality of life for patients diagnosed with depression [10]. However, until now, this has not been feasible, as most studies have identified group-based risk factors rather than within-individual risk factors [11-15]. For example, several studies identified sleep duration as an important predictor of depressive states and depressive episodes in group-based analysis [16,17]. Likewise, there was an association between longer sleep duration and worsening of depressive core symptoms among some individuals, but the causal direction varied between duration of sleep and depression in each individual [18]. Due to the heterogeneity of the course of depression in individuals, intraindividual effects may differ from those found in between-participant studies. This is where n-of-1 studies come into play, as they allow longitudinal testing of the hypotheses within individuals [19].

During the last few decades, digital technologies have remarkably permeated and reshaped the daily lives of people. Globally, there are approximately 6.84 billion smartphone users [20]. To put that into perspective, the figure accounts for around 85% of the 8 billion global population.

Patients can be collectors and owners of large, diverse, long-term datasets through biosensors (wearable devices, including activity trackers), and “biosensor” (short form of biological sensor) data have been defined as physiological measures combined with biological components detected by an analytical device (eg, glucometer, pulse oximeter, and smartwatch) [21]. This raises the question of whether these patient-generated bio- and self-monitoring data can be used for the benefit of patients and systematically integrated into the care processes [22-25].

It has been almost 2 decades since the notion of digital interventions emerged, and efforts have been made to monitor the states of depression and predict them using sensor data. For instance, wearable devices (electronic devices designed to be worn on the body, with the capability to collect, process, and sometimes transmit data) monitor physiological parameters uninterruptedly with high precision [26]. These devices make continual passive sensing easier, that is, recording data of a person with only minimal effort required from their side (eg, to wear and charge the device regularly). Using smartphones and wearable devices may therefore provide an easy and effective way to monitor depressive symptoms, and parameters derived from these monitoring data could serve as digital markers of mood symptoms in depression [27]. Such data could allow further exploration of patterns that are related to the severity and changes in the severity of depressive symptoms.

### Objectives

To our knowledge, numerous systematic and scoping reviews have examined the growing body of research on wearable devices and smartphone-based passive sensing [1,28-37]. However, the included studies were cross-sectional or had short durations (ranging from 1 day to 8 weeks), limiting their ability to capture the long-term course of depressive disorders. Longer studies are better suited for identifying patterns related to symptom severity and its fluctuations over time within the individual. This systematic review aims to fill this gap in the psychiatric and biomedical informatics literature by being the first to focus on longitudinal studies with continuous passive sensor-based monitoring lasting more than 3 months.

Our aim was to systematically review longitudinal studies on patients with depressive disorders and answer the following research questions:

1. Can objective data, automatically collected from wearable devices and smartphones, be used to effectively *monitor* and differentiate between varying states of depression within individuals with a clinical diagnosis of depression?
2. Can objective data, automatically collected from wearable devices and smartphones, be used to *predict* changes in depressive symptom severity within individuals with a clinical diagnosis of depression?
3. Which objective features derived from wearable devices and smartphones are clinically relevant for monitoring and predicting depression within individuals?

## Methods

### Design

This systematic review was conducted based on the guidelines outlined by the PRISMA (Preferred Reporting Items for Systematic Reviews and Meta-Analyses) statement for transparent and comprehensive reporting of the methodology and results [38].

To minimize the likelihood of researcher bias, the search strategy, inclusion criteria, and data extraction were specified and preregistered as an online protocol (PROSPERO registration number: CRD42022355696).

### Search Strategy and Selection Criteria

A preliminary literature search in PubMed was performed using key terms related to depression and biosensors (performed in March 2022). The retrieved articles were used to identify further keywords and build an adequate search string.

We included studies assessing the contribution of available sensor technologies (eg, wearable devices and smartphones) in terms of monitoring and predicting depressive symptoms (depression progression and any fluctuations, ie, improvement or worsening, in symptoms) for a minimum period of 3 months among individuals.

### Inclusion Criteria

The inclusion criteria were as follows: (1) patients belonging to any population with a primary or co-morbid diagnosis of clinical depression (eg, individuals with postpartum or perinatal depression; individuals from the LGBT [lesbian, gay, bisexual, transgender] community; patients with chronic obstructive pulmonary disease, cancer, parkinsonism, and diabetes; students; substance users; individuals with obesity; and smokers); (2) patients from any age group (eg, adolescents [age 14-18 years] and elderly individuals); (3) articles involving any study design (longitudinal, cross-sectional, retrospective, prospective, interventional, observational, etc); (4) studies involving longitudinal monitoring or prediction of depressive symptoms for a minimum period of 3 months; (5) studies published between 2012 and 2022; (6) studies using smartphones, wearable devices, or other sensory devices to assess or monitor clinical depression; (7) studies involving any context, such as geographical location and cultural factors; and (8) studies using any outcome measures for depression.

### Exclusion Criteria

The exclusion criteria were as follows: (1) diary studies (only self-report, no biosensor data collected); (2) non-English articles; (3) opinion pieces, overview articles, reviews, notes, case reports, letters to the editor, extended abstracts, proceedings, patents, editorials, commentaries, conference proceedings, website discussions, blogs, and magazine and newspaper articles; and (4) articles not openly available for full-text reading.

### Definitions

“Smartphone” was defined in line with its MeSH (Medical Subject Headings) term as any phone equipped with a mobile operating system (Android, Apple iOS, Symbian OS, or Windows Mobile) on which apps can be installed to capture data from the phone’s sensors, along with internet connectivity.

“Passive” data collection was defined as data collection without user input or without active participation (eg, collected without disrupting normal activities) [39]. “Active” data collection was defined as data collection through active or conscious participation (eg, journaling or diary technique).

### Literature Search

To collect relevant publications, an electronic database search was conducted. We searched in 2 high-order databases, PubMed (primary database for this work) and Web of Science (also known as Web of Knowledge; secondary database), using Boolean search operators and combinations of keywords of conditions (major depressive disorder and depressive disorder) and technology (smartphone, sensor data, wearable device, mobile phone, smartphone application, sensor, mobile app [only used in PubMed], mHealth, ecological momentary intervention, and digital phenotype). The search was conducted initially on May 17, 2022, and updated on June 7, 2022 (PubMed) and June 9, 2022 (Web of Science). The search term “mobile app” identified a high number of articles that were not matching with the objectives of this review during our search in PubMed. Therefore, we only used “smartphone application” (but not “mobile app”) as a search term during the second search (Web of Science). Using the same search strings, the searches were repeated on February 1, 2023, to include articles from June to December 2022. To identify any further eligible studies and minimize selection bias, an additional search of the reference lists of retrieved articles (pearling) was conducted.

This search identified a total of 12,997 articles. After eliminating duplicate papers (n=112), the initial title and abstract screening took place, and the retrieved papers were screened against the inclusion and exclusion criteria (eg, nonhuman experiments; mobile phone addiction topics; focus on diary methods, which only involve subjective data; and nonmedical-related topics such as bipolar electricity). A total of 49 articles were retrieved for full-text review, and 9 articles met all the requirements for inclusion.

**Figure 1:**
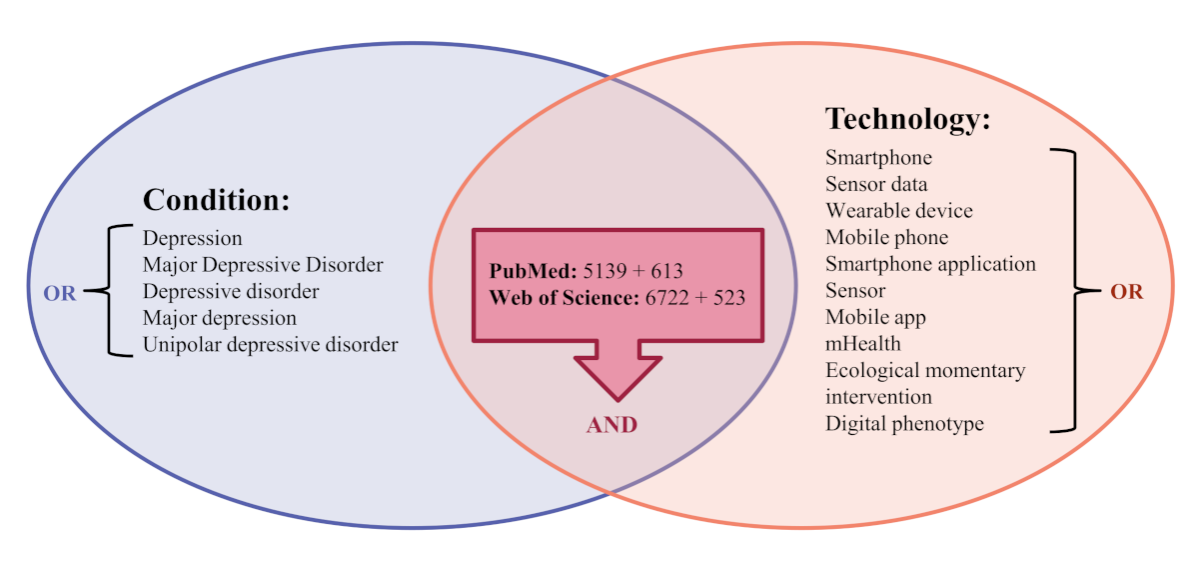
Overview of keyword combinations. PubMed: 5139 (June 7, 2022) and 613 (February 1, 2023); Web of Science: 6722 (June 9, 2022) and 523 (February 17, 2023).

In order to make this search replicable in the future, more details are provided in Table 1 and Figure 1.

**Table 1:** Search details for the PubMed and Web of Science databases.

Search string	Database	Search information (search date, search duration, and hits [N=12,997])
(Depression*[Title/Abstract] OR Major Depressive Disorder*[Title/Abstract] OR Depressive disorder*[Title/Abstract] OR major depression*[Title/Abstract] OR unipolar depressive disorder*[Title/Abstract]) AND (smartphone*[Title/Abstract] OR sensor data[Title/Abstract] OR wearable device*[Title/Abstract] OR mobile phone*[Title/Abstract] OR smartphone application*[Title/Abstract] OR sensor*[Title/Abstract] OR mobile app*[Title/Abstract] OR mHealth[Title/Abstract] OR ecological momentary intervention[Title/Abstract] OR digital phenotype*[Title/Abstract])	PubMed	Date: June 7, 2022; Duration: January 1, 2012, to June 6, 2022; Hits: 5139 Date: February 1, 2023; Duration: June 7, 2022, to December 31, 2022; Hits: 613
(AB=Depression* OR AB=Major Depressive Disorder* OR AB=Depressive disorder* OR AB=major depression* OR AB=unipolar depressive disorder*) AND (AB=smartphone* OR AB=mHealth OR AB=sensor data OR AB=wearable device* OR AB=mobile phone* OR AB=smartphone application* OR AB=sensor* OR AB=ecological momentary intervention* OR AB=digital phenotype*)	Web of Science	Date: June 9, 2022; Duration: January 1, 2012, to June 6, 2022; Hits: 6722 Date: February 17, 2023; Duration: June 7, 2022, to December 31, 2022; Hits: 523

### Study Selection

To control the risk of bias, the PRISMA 2020 recommendations for systematic literature analysis were followed. The PRISMA checklist has been prepared according to the PRISMA 2020 statement (Multimedia Appendix 1) [40]. Studies were independently selected by 3 different authors (RA, SS, and HO), who first analyzed the titles and abstracts and subsequently selected the full papers that met the inclusion criteria, resolving disagreements through consensus. The reasons for rejection were annotated. Subsequently, the authors retrieved the full-text copies of the remaining articles and selected those meeting the inclusion criteria. Disagreements were resolved through discussion and consensus.

### Data Extraction and Synthesis

A systematic extraction form (Microsoft Excel data abstraction sheet) was used for each article to collect the following data: general data (authors, article title, type of publication, year of publication, and journal), study characteristics (aim of the study, study design, main variables of interest, type of electronic device, type of biosensor data, study length, and trial quality), participant data (number of participants, mean age of participants, type of control group, inclusion and exclusion criteria, and dropout rates), and outcome measures (primary outcomes, compliance rates, and missing data).

Data extraction was performed by 3 different authors (RA, SS, and HO), and discrepancies were resolved by internal discussion and majority agreement. Data synthesis was conducted for the extracted data. A narrative synthesis was applied due to the heterogeneity of the extracted data.

### Critical Appraisal

Three independent reviewers (RA, SS, and HO) assessed the quality of all included studies, using Critical Appraisal Skills Program (CASP) checklists [41]. All articles were assessed following CASP questions, which determine if all the required steps for successful scientific reporting are completed and if the relevant information is presented clearly in the selected papers.

## Results

### Study Selection and Flow

**Figure 2:**
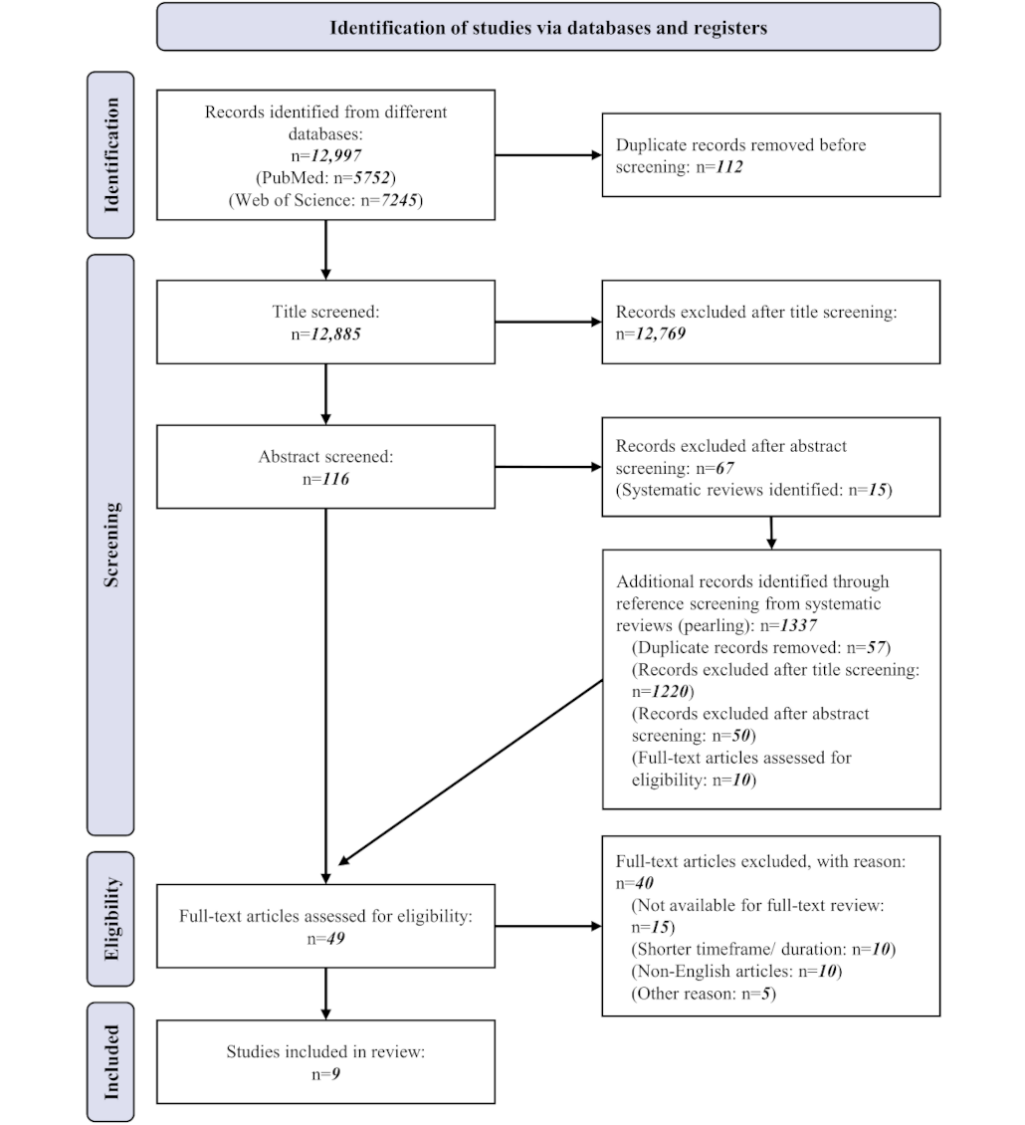
PRISMA (Preferred Reporting Items for Systematic Reviews and Meta-Analyses) flowchart.

The search yielded 12,997 records based on our search parameters across the PubMed and Web of Science databases. After screening the abstracts (n=116), 49 articles potentially eligible for inclusion were retrieved for full-text review. Many studies were excluded due to irrelevance, meaning that their abstracts did not mention the use of wearable technologies or smart devices for the detection of depression, or they did not use wearable devices to measure any physiological parameters. We conducted a further investigation to identify suitable articles through “pearling.” Ultimately, a total of 9 articles were included in the analysis. The most frequent reasons for exclusion were the duration of the study period and the length of continuous data collection [17,42-60]. We collected information about study designs, patient populations, types of digital health interventions, and their intended uses. The PRISMA flowchart is presented in Figure 2. Given the variability in study populations, study designs, and outcomes, we performed a descriptive analysis of the data. Comprehensive details of each study are presented in Table 2.

**Table 2:** Characteristics of the included studies.

Study	Digital technology					Data source			Duration of longitudinal data collection
	Wearable	Smartphone app	Platform	Developer	PROMs^a^		Sensor data		
Tønning et al [61], 2021	No	Yes	Android and iOS	Monsenso	Mood and activity (daily)		Physical activity (steps and total distance), social activity, and smartphone usage (call logs and text messages)	PROMs: average of 130 days/participant; sensor data: average of 158 days/participant (data collection)	
McIntyre et al [62], 2021	No	“mind.me” app	Android	Mind Mental Health Technologies Inc	PHQ-9^b^ (monthly)		Daily call count, daily SMS text message count, location variance, normalized entropy, number of clusters, total distance (km), and mean absolute deviation in distance (km)	12 weeks (study duration)	
Sarda et al [63], 2019	No	Yes	Android	Touchkin	PHQ-9 (biweekly)		Accelerometer, GPS, and ambient light sensor	20 weeks (study duration)	
Fang et al [64], 2021	Fitbit Charge 2	Yes	—^c^	Remedy Health Media LLC	Mood (daily), PHQ-9 (quarterly)		Sleep parameters	Sensor data: average of 115 (SD 111) days/participant (data collection)	
Cho et al [65], 2020	Fitbit Charge HR 2 or 3	“Circadian Rhythm for Mood (CRM)” app; daily “eMoodChart”	Android and iOS	—	Sleep (daily)		Light exposure (Android only), activity, and heart rate	52 weeks (study duration)	
Mullick et al [66], 2022	Fitbit Inspire HR, software version 1.84.5	“AWARE” app	Android and iOS	—	PHQ-9 (weekly)		Heart rate, sleep, steps, calls, conversations, location, Wi-Fi, and screen use	24 weeks (study duration)	
Zhang et al [67], 2021	Fitbit Charge 2 or 3	Several remote technology apps	—	—	PHQ-8^d^ (biweekly)		Sleep, physical activity, stress, speech patterns, and cognitive function	Up to 2 years (study duration)	
Lee et al [68], 2022	Fitbit Charge HR 2 or 3	“eMoodChart”	Android and iOS	Study team	Mood (daily)		Light exposure (Android only), steps, heart rate, and sleep	Average of 279.7 days/participant (data collection)	
Bai et al [69], 2021	Wristband Mi Band 2, Xiaomi Corporation	“Mood Mirror”	Android	—	Mood (daily), PHQ-9 (biweekly)		Sleep data, step count, heart rate, and phone usage (call logs, text messages, app usage, GPS, and screen time)	12 weeks (study duration)	

^a^PROMs: patient-reported outcome measures.

^b^PHQ-9: Patient Health Questionnaire-9.

^c^Not applicable.

^d^PHQ-8: Patient Health Questionnaire-8.

### Study Characteristics

We present a narrative synthesis of the major themes extracted from the 9 studies. This will be followed by a summary of the characteristics of the digital health technologies (DHTs) and a section addressing the findings related to our research questions. Finally, we will summarize key challenges of the approach.

#### Characteristics of the Included Studies

The characteristics of the studies reported in the selected articles are comprehensively presented in Multimedia Appendix 2.

Among the 9 articles, only 1 discussed the results of a randomized study [61]. Eight studies were nonrandomized, single-arm studies [62-69]. Among the studies, 3 assessed the feasibility and validity of DHTs [61,62], while 3 others evaluated the efficacy or effectiveness of DHTs [63-66]. Across the chosen studies, participant recruitment ranged from 45 to 2200 individuals. However, justifications for the chosen sample sizes were seldom provided. The reporting of recruitment strategies was notably concise [61-69]. Study durations varied, ranging from 12 weeks [62] to 1 year [64,65]. Either the study completion rate or adherence to the technology was reported. Geographically, 4 studies originated in Asia [63,65,68,69], 2 in Europe [61,67], and 3 in North America [62,64,66]. Among these, 2 were multisite studies (different countries) [62,67]. The study by Sarda et al [63] mentioned providing data recharge (1 GB) per month to cover usage costs without any additional incentives. In the remaining studies, information concerning economic compensation for participation was not provided.

#### Characteristics of Patient Populations

All studies included patients diagnosed with major depressive disorder and unipolar depressive disorder according to ICD-10. Female participants consistently outnumbered male participants in all studies [61-69]. All studies enrolled adults aged >18 years [61-65,67-69], except for 1 study that included adolescents aged 14-18 years [66]. One study recruited participants from residency training [64], while the remaining studies included participants from diverse backgrounds.

#### Summary of Collected Data

The studies included in the analysis used the Patient Health Questionnaire-8/-9 (PHQ-8/-9) [62-66,69] and Beck Depression Inventory-Second Edition [61] self-report instruments as principal outcome measures. There were variations in the timing of outcome assessment (daily [61,64,65,68,69], weekly [66], biweekly (every 2 weeks) [63,67,69], monthly [62], and quarterly [64] assessments).

In addition to self-ratings of depressive symptoms, the most commonly collected data included the number of steps per day [61,68] and the total distance moved per day [61,62], which were based on GPS [63,69], Wi-Fi signals [66], and mobile cell towers. Smartphone usage metrics, such as total screen-on time per day and the number of times the screen was turned on per day, were also collected [66].

Moreover, the studies gathered data on device analytics, encompassing call logs (daily call count and the duration of calls per day) [66,69] and SMS text message patterns (daily frequency). One study also incorporated accelerometer and ambient light sensor data into the data collection efforts [63].

### Characteristics of DHTs

#### Electronic Devices and Use of Sensors

The DHTs evaluated across the studies could be broadly classified into 2 categories: mobile or smartphone apps and wearable devices. In certain studies, smartphone apps were employed to collect patient data in real time, coupled with daily mood and symptom monitoring through self-reports [61-64,66,68,69]. In 6 studies, wearable devices, such as Fitbit Charge, were used in conjunction with smartphone apps to passively accumulate activity data [64-69]. One study used daily push notifications at a specific time to assess mood [64]. Another study implemented SMS text message reminders if participants missed mood recording sessions [65].

#### Operating Systems

Four studies used the Android operating system [62,65,68,69], while another study used both Apple iOS and Android [61]. The operating system used in the remaining studies could not be ascertained.

#### Data Processing and Use

All studies conducted time-series analysis. Among them, 3 studies conducted comparisons within individuals [61,66,67], while the other studies conducted comparisons between individuals and between groups [62-65,68]. Complex calculations, such as data classification and prediction modeling, were performed in all studies, but not directly on the smartphone. For some studies, it could not be determined whether a remote server was used [61,62,64]. In 7 studies, data were processed and correlated with clinical self-rating scales for depression as validation measures to test the validity of interpretations made through passive sensing [61-64,66,67,69]. The correlation analysis was typically performed after the study’s completion. Various families of algorithms were employed for data processing to interpret or predict participant status in different studies.

#### Usability and Effectiveness of Health Technologies

In 1 study, user satisfaction with the app was assessed using a qualitative questionnaire, and all respondents who completed the questionnaire indicated a high level of satisfaction with the app [62]. Overall, the selected studies indicated high feasibility and acceptability of technologies for mental health care.

### Synthesis of Findings Related to the Research Questions

To summarize, of the 9 studies, 1 (11%) was able to discriminate among different states of depression (worsening, relapse, or recovery) and 6 (67%) reported prediction capabilities for depressive episodes and symptom severity. Detailed descriptions of the results regarding the 3 research questions of this systematic review are presented below.

#### Passively Collected Data for Monitoring Depressive Symptom Severity

The primary intended use of digital interventions in the selected studies was the monitoring of depressive symptoms to gain an understanding of disease progression and clinical assessment [61]. For monitoring purposes, different parameters were collected through sensors by different studies (eg, call logs [61,62,66,67,69], step count [61,63,66-69], GPS data [61-63,66,69], smartphone usage [61,69], light sensor data [63,65,68], sleep [64-69], and heart rate [65,66,68,69]).

Discriminating among different states of depression (worsening, relapse, or recovery) was found to be feasible in 1 study [64]. In the study by Zhang et al [67], daily sleep records of participants were collected using the Charge 2 or Charge 3 device (Fitbit Inc), alongside the variability of each participant’s depressive symptom severity measured through the PHQ-8. The study demonstrated that 5 sleep features (sleep architecture, sleep stability, sleep quality, insomnia, and hypersomnia) exhibited a significant relationship (*P*<.001) with the worsening of depressive status over the following 2 weeks [67].

#### Passively Collected Data for Predicting Changes in Depressive Symptom Severity

The capability of passively collected data for predicting depressive episodes and states was shown by 6 studies [62,63,65,66,68,69].

In 1 study, a model was trained using user PHQ-9 scores and phone usage data, including call logs, SMS data, and GPS information [62]. The study illustrated a predictive accuracy of 0.91 (SD 0.06) for identifying individuals with clinically significant depressive symptoms, accompanied by a sensitivity of 0.98 and specificity of 0.93. Notably, phone usage features were extracted from data collected 14 days before the assessment of the PHQ-9 [62].

Sarda et al [63] collected a total of 53 passive sensing variables derived from the activity, mobility, sleep, and communication data from smartphone sensors (aggregated daily) and PHQ-9 data biweekly. Subsequently, using these derived sensing variables, a classification predictive model using a gradient boosting machine learning classifier was constructed. This model demonstrated the best performance, achieving a cross-validation accuracy of 79.07% (95% CI 74%-84%) and a test accuracy of 81.05%. Daily predictions were conducted for 950 days (with missing features in one or more of the 53 derived sensing variables) out of 2694 instances [63].

The study conducted by Cho et al [65] employed a mood prediction feedback model using a smartphone app called Circadian Rhythm for Mood. This model used machine learning to analyze real-time acquired personal digital phenotypes, including factors such as heart rate, activity, sleep, and light exposure. The mood prediction was visually conveyed to users through a facial expression icon (emoticon), providing an intuitive indication of how their mood was predicted to change over the following 3 days. Users received this daily visual feedback when they activated the app. However, the study did not specify the predictive accuracy achieved [65].

Mullick et al [66] conducted a study aiming to predict depression scores and changes in depression levels among adolescents, with the intention of understanding how sensor features contribute to this prediction. Passively sensed data from mobile phones were collected through the AWARE app, alongside data from a wearable device (Fitbit Inspire HR). The researchers applied regression-based machine learning algorithms to forecast depression scores based on weekly PHQ-9 surveys. The personalized models, which demonstrated superior performance in predicting depression in comparison to universal models, yielded the best outcomes. These models were constructed from feature sets that incorporated data from Fitbit, location, calls, and screen usage. Remarkably, this personalized model achieved the prediction of depression scores and weekly changes in depression with root mean squared errors of 2.83 and 3.21, respectively [66].

Lee et al [68] used random forest (a supervised learning algorithm) to train the episode prediction model. By selecting features, a set of optimal parameters (circadian rhythm–related digital phenotypes: light exposure, steps, and sleep) was identified and used for predicting whether a mood episode would occur within the next 3 days. They showed that features that contributed to increased risk of forthcoming episodes were lower mean sleep efficiency, higher standard deviation of sleep length, higher mean step count during bedtime, and lower mean step count during afternoon and evening, with a predictive accuracy of 87% for model-predicted mood episodes compared with predictions made as a result of clinical interviews [68].

Another study assessed the prediction of mood changes (assessed through biweekly self-rating via the PHQ-9) by selected features collected through the Mood Mirror app and a wristband (call logs, sleep, step count, and heart rate) [69], and an accuracy rate of 84.27% was obtained.

#### Machine Learning Approaches Used for Prediction

The reviewed studies share a common goal of leveraging passive sensor data and machine learning to predict depressive symptoms and mood fluctuations. Despite differences in data sources, modeling strategies, and clinical objectives, they provide valuable insights into the role of digital phenotyping in mental health prediction. Table 3 summarizes the analytical approaches that were applied in the selected studies.

**Table 3:** Summary of the analytical approaches and algorithms.

Study	Goal^a^	Main findings	Data used	Algorithms used	Key results
Tønning et al [61], 2021	—^b^	Smartphone usage data and clinical depression measures are associated in unipolar depressive disorder.	Inputs: automatically generated smartphone data (steps, phone usage, calls, and screen time) and self-reported data Outputs: depressive symptom severity (Hamilton Depression Rating Scale score)	Inferential statistical tests (eg, *t* tests and chi-square) for analyzing associations between automatically generated smartphone data and clinical depression measures.	Association analysis showed links between phone usage (calls, steps, and screen time) and depressive symptoms. Lower psychosocial functioning was associated with fewer daily steps (*P*=.04) and increased phone calls (*P*<.05).
McIntyre et al [62], 2021	P	Sensor data show strong convergence with PHQ-9^c^ depression scores in adults with depressive symptoms.	Inputs: mind.me app sensor data (GPS location, SMS text message logs, call logs, and behavioral data) Outputs: depressive symptom severity (PHQ-9)	Multimodal data from GPS, SMS text messages, and call logs were processed using statistical models. Machine learning models for convergence analysis between sensor data and PHQ-9 depression scores.	The predictive algorithm calculated sensitivity (0.98) and specificity (0.93) while achieving an overall predictive accuracy of 0.91 (SD 0.06) for detecting depressive symptoms. User satisfaction with the app was high, and convergence with the PHQ-9 was statistically significant.
Sarda et al [63], 2019	P	Smartphone sensing for activity, mobility, and social interaction predicts depressive symptoms in patients with diabetes.	Inputs: smartphone-sensed data (activity, mobility, sleep, and communication patterns) Outputs: depressive symptom severity (PHQ-9)	Supervised learning approach applied to features derived from smartphone-sensed data. XGBoost classifier used for the prediction of depression symptoms. Model performance was measured using cross-validation and test set accuracy.	The XGBoost model achieved 81.05% accuracy in identifying depression symptoms in patients with diabetes. Cross-validation accuracy was 79.07% (95% CI 74%-84%). Significant predictors included average activity rate (*P*=.005) and call activity (*P*<.001).
Fang et al [64], 2021	—	Sleep variability significantly affects mood and depressive symptoms in medical interns.	Inputs: wearable device data (sleep duration, bedtime, wake time, total sleep time, and sleep variability) Outputs: depressive symptom severity (PHQ-9) and daily mood	Linear regression for analyzing the effects of sleep variability on mood and depressive symptoms.	Beta coefficients for total sleep time (b=–0.11; *P*<.001), bedtime (b=0.068; *P*=.02), and sleep variability (b=0.4; *P*=.001).
Cho et al [65], 2020	P	A smartphone app with wearable device tracking reduces mood disorder episodes and improves patient health behaviors.	Inputs: wearable device data (circadian rhythm features) Outputs: prediction of mood episode recurrences (frequency, duration, and type of episode)	Machine learning model embedded in the CRM^d^ app, using wearable device data to predict and prevent mood episodes. The feedback system used a prediction model based on circadian rhythm features, which triggered alerts for behavioral correction.	The CRM group had 97.4% fewer mood episodes and 98.9% shorter episode durations than the control group. The number of mood episodes was significantly reduced (exp β=0.026; *P*=.008), and the duration of mood episodes (exp β=0.011; *P*<.001) also decreased significantly.
Mullick et al [66], 2022	P	Passive smartphone and wearable device sensors predict adolescent depression using personalized machine learning models, which outperform universal models.	Inputs: passive sensor data from smartphones and Fitbit (call logs, conversation, location, and heart rate) Outputs: depressive symptom severity (PHQ-9)	Machine learning models using both linear (eg, linear regression) and nonlinear algorithms (eg, support vector regression) to predict PHQ-9 scores and weekly change in depressive symptoms. Personalized modeling strategies were applied using methods like “leave-one-out” and “accumulated weeks” approaches.	Personalized machine learning models achieved a root mean square error of 2.83 for PHQ-9 score prediction and 3.21 for weekly change in depression severity. Personalized models outperformed universal models.
Zhang et al [67], 2021	M	Sleep features extracted from consumer wearable devices are associated with depressive symptom severity.	Inputs: Fitbit sleep data (18 sleep features, eg, sleep quality, insomnia, and hypersomnia) Outputs: depressive symptom severity (PHQ-8e)	Linear mixed-effects models for analyzing the relationship between wearable device–derived sleep features and the PHQ-8. Statistical coefficients (*z*-scores) were computed to measure the effect sizes of features such as insomnia, hypersomnia, and sleep stability.	Linear mixed regression identified 14 significant sleep features, including awake time percentage (*z*=5.45; *P*<.001), awakening times (*z*=5.53; *P*<.001), insomnia (*z*=4.55; *P*<.001), mean sleep offset (*z*=6.19; *P*<.001), and hypersomnia (*z*=5.30; *P*<.001).
Lee et al [68], 2022	P	Digital phenotypes related to circadian rhythms predict impending mood episodes in depressive and bipolar disorder with high accuracy.	Inputs: digital phenotypes from wearable devices and smartphones (circadian rhythm parameters) Outputs: prediction of mood episode occurrences (clinical interview)	Machine learning models (supervised classification algorithms) to predict mood episodes using circadian rhythm parameters.	Prediction accuracy for mood episodes: depressive (90.1%), manic (92.6%), and hypomanic (93%) episodes. AUC^f^ values for the mood episode prediction models: 0.937, 0.957, and 0.963, respectively.
Bai et al [69], 2021	P	Machine learning models using passive data from smartphones and wearable devices predict mood stability in patients with major depressive disorder.	Inputs: passive data from smartphones and wearable devices (call logs, sleep data, step count, and heart rate) Outputs: mood stability states (steady vs swing) labeled from the PHQ-9	Multiple classification models were trained on the data, using various combinations of data to classify mood stability into steady or swing states. Feature selection models were applied to identify relevant features, and 6 machine learning models were compared.	Prediction of steady versus mood swing states achieved 76.67% accuracy and 90.44% recall. The classifier predicted mood swings using call, sleep, step count, and heart rate data, with 252 features extracted for model training.

^a^Studies aiming to monitor depression are labeled with M, and studies aiming to predict depression are labeled with P.

^b^Not applicable.

^c^PHQ-9: Patient Health Questionnaire-9.

^d^CRM: Circadian Rhythm for Mood.

^e^PHQ-8: Patient Health Questionnaire-8.

^f^AUC: area under the curve.

All studies integrated passive sensing data (eg, sleep, heart rate, activity, and smartphone usage) with self-reported measures (eg, PHQ-9, PHQ-8, and mood scores obtained through ambulatory assessment). While Fang et al [64], Zhang et al [67], and Sarda et al [63] focused on sleep and activity data from wearable devices, studies like those by Mullick et al [66] and Bai et al [69] enhanced predictive accuracy by incorporating phone usage patterns (eg, calls, messages, and app activity). The studies differ in predictive targets, with some estimating continuous depressive severity scores [66,69] and others classifying mood states [69] or predicting episode recurrence [65,68], influencing model selection and applicability.

Linear regression was used in studies, such as those by Fang et al [64] and Zhang et al [67], to establish statistical associations between sleep metrics and depressive symptoms. In contrast, more advanced machine learning models, such as XGBoost [63] and support vector regression [66], achieved higher predictive performance but sacrificed interpretability. Personalized modeling approaches [66,69] outperformed generalized models [64,67] by leveraging individual-specific baselines, improving prediction accuracy but requiring additional training data per user. Cho et al [65] took an intervention-driven approach, integrating real-time mood prediction and behavioral feedback loops to reduce depressive episode recurrence.

A key methodological divergence lies in predictive objectives. While some studies estimated continuous depression severity, others classified mood transitions or predicted episode recurrence. This variation impacts how success is measured: classification models [68,69] support binary decision-making, whereas regression models [66,67] offer a more granular understanding of mood trajectories. Furthermore, explainability remains a challenge; linear models provide interpretable relationships between predictors and outcomes, while complex models (eg, XGBoost) optimize accuracy at the cost of transparency, potentially limiting their clinical adoption. Unfortunately, none of the studies assessed the reliability of their predictions over time.

#### Relevance of Individual Biosensor Data

All studies showed the potential of passive sensing using smartphones. The findings encompassed significant correlations with validation measures and the successful construction of prediction models for some or all of the variables studied. However, certain sensor data, such as sleep features (total duration and wake-up time), and other data, such as social contact (call logs) and activity (step count and GPS), could potentially serve as significant parameters for clinical aspects related to depression, including relapse or depressive symptoms.

For example, Zhang et al [67] identified 14 sleep features that exhibited a significant association (*P*<.05) with depression status. Within this set, 5 sleep features, namely sleep architecture, sleep stability, sleep quality, insomnia, and hypersomnia, demonstrated a significant (*P*<.001) relationship with the deterioration of depressive symptoms over the subsequent 2 weeks. Among these sleep features, the percentages of light sleep, nonrapid eye movement sleep, and sleep efficiency were significantly and negatively associated with the PHQ-8 score. Conversely, the remaining significant features displayed a positive association with the PHQ-8 score.

The study by Tønning et al [61] revealed a significant association between patient-reported mood and activity and automatically generated smartphone data. For instance, lower reported mood was linked to a lower step count (*P*=.04) and an increased number of incoming (*P*=.03) and outgoing (*P*=.02) calls, missed calls (*P*=.007), and longer phone calls (*P*=.01) [61].

Univariate analysis conducted in 1 study revealed a significant difference in the average activity rates (*P*<.001), the number of screen-on times at night (*P*<.001), and the average total number of calls (*P*<.001) among individuals exhibiting symptoms of major depression [63].

Fang et al [64] demonstrated that, within individuals, an increase in total sleep time (*P*<.001), a later wake time (*P*<.001), and an earlier bedtime (*P*<.001) were associated with improved mood on the following day.

The study conducted by Mullick et al [66] demonstrated that individuals experiencing depressive symptoms exhibited a tendency to be less active in terms of movement, a behavior that could be captured through location (GPS) data. Furthermore, depression led participants to decrease their interactions with friends and family, as indicated by call-related features. Other studies similarly identified significantly lower levels of social contact (call logs) and reduced daytime activity among individuals reporting symptoms of depression (PHQ-9 score >9) [61,63].

### Critical Appraisal of Study Quality

Quality appraisal was performed taking into consideration 9 domains of research paper quality, including research aims, research design, recruitment strategy, data collection and analysis methods, ethical issues, outcome measures, etc [41]. The results are summarized in Table 4. The quality appraisal showed overall consistent quality across studies for the domains of research aims, research design, recruitment strategy, data collection and analysis methods, and ethical issues. However, there were some quality considerations that must be particularly noted, for instance, the 5th domain addressing the considerations between the researcher-participant relationship was rather scarce. In fact, there was no sufficient description of such rapport across the retrieved studies. Moreover, the value of research assessed in the 9th domain did not consist of the precise parameters that can be followed to assign the value of particular research within the field, therefore relying on the subjective understanding of the author rather than a quantitative unit of measurement.

**Table 4:** Critical Appraisal Skills Program (CASP) checklists and assessment for each study.

Research question	Study								
	Tønning et al [61], 2021	McIntyre et al [62], 2021	Sarda et al [63], 2019	Fang et al [64], 2021	Cho et al [65], 2020	Mullick et al [66], 2022	Zhang et al [67], 2021	Lee et al [68], 2022	Bai et al [69], 2021
1. Was there a clear statement of the aims of the research?	Yes	Yes	Yes	Yes	Yes	Yes	Yes	Yes	Yes
2. Was the research design appropriate to address the aims of the research?	Yes	Yes	Cannot tell	Yes	Yes	Cannot tell	Yes	Yes	Yes
3. Was the recruitment strategy appropriate for the aims of the research?	Yes	—^a^	Yes	Yes	Yes	Yes	Yes	—	Yes
4. Was the data collected in a way that addressed the research issue?	Yes	Yes	Yes	Yes	Yes	Yes	Yes	Yes	Yes
5. Has the relationship between the researcher and participants been adequately considered?	No	—	Cannot tell	No	—	Cannot tell	—	Yes	No
6. Have ethical issues been taken into consideration?	Yes	Yes	Yes	Yes	Yes	Yes	Yes	Yes	Yes
7. Was the data analysis sufficiently rigorous?	Yes	No	Yes	Yes	Yes	Yes	Yes	Yes	Yes
8. Is there a clear statement of the findings?	Yes	No	Yes	Yes	Yes	Yes	Yes	No	Yes
9. How valuable is the research?	Valuable for the research field	Valuable for the research field	Valuable for the research field	Valuable for the research field	Valuable for the research field	Valuable for the research field	Valuable for the research field	Valuable for the research field	Valuable for the research field

^a^Not applicable.

### Key Challenges

#### Advantages of Passive Sensing

Only 1 study reported providing feedback to study participants [65]. Feedback messages were automatically generated and displayed on the app screen to assist patients in modifying their behavior. In addition, warning alerts were conveyed to patients through SMS text messages with a message reading, “Recently, your life rhythm is irregular.” This study computed data locally and delivered feedback directly on the phone. The analyses conducted in this study revealed significant positive behavioral changes after participants received warning alert feedback (*P*<.05) in relation to light exposure during the daytime and steps taken during the daytime. Most of the advice given in the warning alert pertained to increasing physical activity. However, concerning sleep behavior, it was observed that sleep efficiency and duration tended to increase following the point at which the warning alert was delivered.

The study conducted by Cho et al [65] observed the prognosis over a period of 1 year, contingent upon whether the intervention group incorporated feedback alongside conventional treatment. This study identified a robust recurrence prevention effect within the intervention group. Additionally, the study noted a significantly shortened duration of mood episodes and a quicker recovery time subsequent to the recurrence of mood episodes among participants in the intervention group (univariate general linear analysis identified 60.7% fewer total depressive episodes [*P*=.03] and 48.5% shorter depressive episodes [*P*<.001], and multivariate general linear analysis identified 96.7% fewer total depressive episodes [*P*=.03] and 99.5% shorter depressive episodes [*P*<.001]) [65].

#### Challenges of Passive Sensing

The apparent ease of deploying passive sensing was counter-balanced by several reported challenges. Although not systematically reported across studies, these challenges could be divided into 3 categories: technological, methodological, and privacy issues.

Regarding technological challenges, 1 retrieved study mentioned that battery life affected participants’ adherence to Fitbit [66]. Regarding methodological challenges, 1 study noted concerns about generalizability due to a low sample size, possible sample bias, and variability in the study sample data [65]. Regarding the protection of privacy, while all studies did not extensively discuss or explicitly mention privacy concerns, they generally described their methods for safeguarding data privacy. These methods included secure communication with external servers, data anonymization, and audio scrambling [61-69].

#### Dealing With Missing Data

One of the significant challenges to address is the issue of missing data. Adherence rates were reported in only 2 studies [65,66]. The occurrence of missing data and the strategies employed to handle it were rarely outlined. Furthermore, none of the studies referenced a previously published protocol. The included studies exhibited poor reporting of missing data, both at the sample level (eg, study noncompletion) and the individual level (eg, nonadherence).

In the study by Sarda et al [63], participant-day instances with missing values in one or more of the 53 derived sensing variables were removed, resulting in 950 out of 2694 instances available for analysis. Another study reported that the passive sensing data and Fitbit data were on average 69.11% and 32.36% complete, respectively, while PHQ-9 surveys were completed 69.01% of the time [66]. Zhang et al [67] only included datasets with a completion rate of over 85% [67]. In the study by Lee et al [68], participants who wore wearable devices for at least 30 days were included in the analysis, which constituted 54.5% of the original cohort.

Missing data can arise from technological issues, such as device and system failures, or from user-related problems that might be associated with depressed mood [67]. Identifying the underlying reasons for missing data and addressing them in terms of either exclusion or analysis are crucial. Furthermore, thresholds for defining what constitutes missing data varied across the studies.

#### Discontinuation

Reasons for dropout were provided in 1 study and were attributed to the unwillingness to participate in a noncompensated study for a period of 12 weeks [62]. Other studies did not provide specific information regarding discontinuation.

## Discussion

### Principal Findings

Developing and implementing new tools for the self-management and prevention of depression are priorities due to its high prevalence and recurrent nature [32,70-72]. However, a significant gap remains between research and clinical practice. This study included 9 original articles and systematically reviewed evidence on objectively measured data from smartphones and wearable devices used to monitor depression for at least 3 months. To our knowledge, this is the first review to assess the capabilities of these technologies for monitoring and predicting depressive symptoms over an extended period, introducing a lifetime perspective into the field.

Only 1 study in this review addressed whether different depression states (eg, worsening, relapse, or recovery) can be distinguished using sleep data from a wearable device and linear mixed-effects models [67]. Most other studies examined general associations between objective features and depression [61,64] or focused on symptom or episode prediction (see the discussion of the principal findings regarding research question 2 below), rather than real-time monitoring. This shows that behavioral patterns and physiological changes, which may precede conscious emotional awareness, remain underutilized for assessing depression severity. Because self-perception often lags observable symptoms or physiological shifts, individuals may recognize their need for help only when the condition has already worsened. Objective markers from smartphones and wearable devices could help bridge this gap and support the timely detection and monitoring of depressive disease states. While following contemporary diagnostic manuals [73,74], depression is primarily defined by affective and cognitive symptoms, such as low mood, anhedonia, fatigue, and hopelessness. These rely on self-report and are not directly measurable by passive sensing (or other biomarkers [75]). In contrast, behavioral and physiological domains, also characteristic of depression, offer more promise for objective, continuous monitoring and should be explored further in future research.

Six studies demonstrated that objectively measured data can predict depressive episodes or symptom severity. Four of these studies reported predictive accuracies for depression measures based on data collected via smartphones and wearable devices (predictive accuracy ranged from 81% to 91%) [62,63,68,69]. Three studies predicted depressive symptom severity assessed by the PHQ-9 (biweekly; predictive accuracies: 81% [63], 84% [69], and 91% [62]). Higher predictive accuracies were achieved in studies with a personalized modeling approach [69] or those that were using multimodal data streams as model input [62]. Although these results are promising, the long-term prediction of depression using sensor data from smartphones and wearable devices faces several potential limitations. Depression is highly heterogeneous, and generic models may fail to capture person-specific symptom trajectories over time. However, only 1 study applied personalized modeling [66], leaving space for exploring idiographic approaches in future studies. Our review focused on longitudinal studies investigating depression from a lifespan perspective. However, models trained on short-term datasets (eg, 12 weeks [62,69]) may not generalize well over longer periods. As health-related behaviors and associated symptoms evolve over time, past data may no longer reflect current patterns, and model accuracy would be reduced. Technical issues (eg, sensor malfunction and inconsistent data sampling) or a lack of user engagement and compliance (eg, not wearing a device and disabling tracking) can lead to data attrition. Missing data can compromise prediction quality, but only 1 of the included studies [66] reported both adherence rates and handling of missing data during analysis.

The objective features most commonly examined in the reviewed studies and significantly associated with depression were primarily behavioral in nature, including activity [61,63,66,69], sleep [64,66,67,69], and smartphone usage [61,63,66,69]. Several studies reported significant associations between depression and physiological features obtained from wearable devices, including heart rate [66,69] and circadian rhythm [65,68]. This review could not determine whether single features are clinically relevant for monitoring and predicting depression within individuals, as the methodological heterogeneity across studies prevented reliable and generalizable conclusions. Even when feature importance was reported, it was specific to the analytical methods used, limiting the ability to draw conclusive comparisons across studies.

### Comparison With Prior Work

Traditionally, semistructured interviews complemented by self-report measures have been used as diagnostic procedures, capturing a static moment in time. A few former valuable reviews in a similar field provided the groundwork for our review. For example, a systematic review by Köhnen et al [32] included studies with durations ranging from 1 to 52 weeks and using offline computer programs, mobile texts, or videoconferencing tools as a medium of intervention [32]. Two other reviews only included studies with younger populations and adolescents [34,35]. Another review included only randomized controlled trials [76]. Owing to recent advancements in sensor technologies, physiological signals can now be recorded unobtrusively using devices like smartwatches and smartphones. This approach does not entail recording sensitive information (unlike cameras and audio signals), and the associated sensors do not disrupt users’ daily routines. Consequently, patient-specific models can be automatically generated to continuously estimate the patient’s affective state [71,77]. Certainly, we agree that self-reports play a pivotal role in monitoring depressive symptoms, given that objective observations might not always align with patients’ subjective experiences. Nonetheless, our study encompassed research that gathered self-reports along with data collected from wearable biosensors or smartphones, and applied machine learning techniques to the acquired data whenever feasible.

From the results, it is clear that monitoring and predicting depressive symptoms through digital phenotyping can be considered worthy measures for improving the quality of life of patients with depression. The existing evidence indicates that the use of passively collected data could potentially lead to enhanced management of depressive symptoms [61-69]. For instance, Tønning et al [61] conducted a study that identified correlations between automatically generated data and daily self-reports. This information could subsequently be employed to implement just-in-time adaptive interventions, which involve providing smartphone-based treatments when machine learning models predict instances of depressed mood. While the studies under consideration did not uncover any notable associations between specific biosensor data and relapse, these findings can serve as a foundational stepping stone for further exploration in this domain. Three studies passively collected GPS and location data [61-63,66], which can be considered very sensitive information. Therefore, it is crucial to address ethical concerns pertaining to privacy, consent, and self-awareness. While individuals might be open to sharing personal data for research purposes, they could exhibit greater caution when commercial interests come into play [30,78,79]. Additionally, it is argued that the measurement of distance traveled by smartphone-based GPS receivers might differ [80,81]. Similarly, accelerometers are commonly considered reliable, but variations in their output and validity in measuring physical activity have been noted [80,82].

Digital interventions have faced criticism due to concerns about them potentially exacerbating anxiety through continuous monitoring and impeding the enjoyment of physical activities [83,84]. However, despite these critiques, the integration of wearable devices and smartphones with feedback mechanisms has shown potential in effectively alleviating symptoms and could contribute to relieving the burden on patients by managing their episodes and consequently mitigating the burden on clinicians [1,65]. For individuals experiencing milder levels of depression, smartphone apps could potentially serve as an excellent self-management tool. Doherty et al [85] demonstrated that user engagement is also influenced by the severity of depression; however, the specific direction in which this influence operates remains to be clarified [76]. Furthermore, the studies examined in this review concentrated on a limited set of predictor variables. Consequently, further research involving the analysis of multiple variables over an extended duration will be essential to identify significant predictors for the relapse of depressive episodes. While assessing socialization, call logs were taken into account. However, features related to proximity using Bluetooth and microphone sensors appear to be more attuned to mood than mere counts of calls and messages. This presents a novel avenue for exploration and warrants further investigation [80].

### Strengths and Limitations

Our review was conducted in accordance with the PRISMA guidelines [38], reflecting rigorous methodological standards. Our broad search strategy identified 12,997 peer-reviewed articles focused on approaches to support mental health. This substantial number revealed the rapid evolution of the literature in this domain, which has grown considerably larger compared to smartphone interventions for other health conditions [72]. Furthermore, we used a comprehensive search term to ensure the inclusion of all pertinent articles and minimize the risk of overlooking relevant information.

Nevertheless, we must acknowledge certain limitations. First, we may have overlooked eligible trials due to different reasons: (1) studies may have been published in languages other than English; (2) eligible studies exclusively published in technology-oriented journals could have been inadvertently omitted, as our literature search was limited to databases specializing in medical and psychological publications; and (3) the search terms and selected keywords did not cover all potential studies. Due to the nascent stage of the research field, MeSH terms have not yet been fully established, presenting challenges to the consolidation of knowledge across the research field. Second, due to the absence of standardized evaluation and reporting practices, comparing technologies across studies posed a significant challenge. This review included studies that used diverse methods for diagnosing depression, making it challenging to establish strict inclusion criteria concerning diagnostic assessments [61-69]. For instance, variations were observed in basic descriptors of sample characteristics, recruitment methods, data collection durations, depression assessment measures, the handling of missing data, the types and quantities of sensors employed, the timing and location of data processing, and the manner in which feedback was provided to users. Most importantly, retrieved studies were not uniform in terms of data collection (both self-reported and sensor data). The types of sensors used (ie, wearable devices and smartphone sensors) also varied. These differences make it difficult to reliably compare parameters. Third, the selected studies adhered to the criterion of selecting adults aged 14 to 65 years. In future research, it will be essential to assess functionality (in terms of monitoring and prediction) in a population that better reflects the diversity of the real world, thereby enhancing the generalizability of the findings. Among the chosen studies, only the study by Fang et al [64] used education level as a selection criterion; unfortunately, no specific information was provided regarding the education levels of participants in the other studies. This factor could potentially influence the use of and openness to health technology [86]. Fourth, the duration of the studies included in this review varied, with the continuous data collection period typically being relatively short (averaging around 3 months). As a result, there was insufficient data available to assess the long-term usage of passive sensing DHTs for mental health. While the outcomes observed in the short term are encouraging, it remains uncertain whether the substantial volume of data derived from DHTs can truly revolutionize mental health care and enhance patient outcomes over their lifespan.

### Future Directions

To enhance long-term self-management practices for depressive symptoms, the provision of personalized feedback based on passively sensed data would be advantageous. Patient adherence to any intervention for self-management plays a very crucial role, and this needs to be addressed in future studies. Upcoming studies should undertake comprehensive assessments of the acceptance, potential adverse outcomes, and longitudinal use of passive sensing systems, given their expanding technological reach.

All studies that used passive data for the prediction of depression collectively highlighted the promise of digital biomarkers and machine learning in mental health prediction, but differences in target populations, modeling approaches, and predictive outputs restricted direct comparability. Personalized models demonstrate strong predictive performance but require individualized calibration, whereas generalized models are more scalable but risk lower accuracy for specific users. The trade-off between model interpretability and predictive power remains a challenge, particularly for clinical integration. Moving forward, standardized benchmarks, clearer definitions of predictive targets, and transparent reporting on feature selection and performance trade-offs are essential to improve the comparability and real-world applicability of predictive models in mental health care.

This review highlighted significant heterogeneity in the configurations of data collection, reporting, and processing. These disparities emphasize the urgency of adopting a more consistent adherence to reporting guidelines that have already been established in closely related fields [87]. Based on our clinical and research experience, we make the following recommendations to enhance the reporting and generalizability of research concerning passive sensing in patients with depression. Regarding the study population, studies should (1) provide more precise reporting of recruitment strategies to increase the scope of reproducibility; (2) present basic demographic and clinical data, including age, gender, ethnicity, and comorbidities; and (3) report more clearly on user engagement and acceptability. Regarding data collection and analysis, studies should (1) use validated scales for assessing depression; (2) preregister the study protocol to increase reliability; and (3) offer a clear description of missing data management.

### Conclusion

Our review demonstrated the potential of longitudinal passive data from smartphones and wearable devices for the prediction of depressive symptoms among individuals. However, few studies have addressed their potential for illness monitoring. Passive sensing via wearable devices and smartphone apps is in harmony with the principles of minimally disruptive medicine and may help to identify the early progression of symptoms. Predicting individual illness trajectories ahead of time facilitates early interventions like self-management strategies that could be offered by smartphone apps or adjusting traditional therapeutic interventions for depression to the intensity and frequency that is needed in a specific moment for each individual.

## Appendix

Multimedia Appendix 1 PRISMA checklist.

Multimedia Appendix 2 Characteristics of the included studies.

## Abbreviations

CASP: Critical Appraisal Skills Program
DHT: digital health technology
ICD-10: International Classification of Diseases, version 10
MeSH: Medical Subject Headings
PHQ-8: Patient Health Questionnaire-8
PHQ-9: Patient Health Questionnaire-9
PRISMA: Preferred Reporting Items for Systematic Reviews and Meta-Analyses

## Data Availability

Retrieved data are provided as a supplementary file, and all retrieved articles and generated data are available upon request from the corresponding author.

## References

[1] Hickey BA, Chalmers T, Newton P, Lin C, Sibbritt D, McLachlan CS, Clifton-Bligh R, Morley J, Lal S (2021). Smart devices and wearable technologies to detect and monitor mental health conditions and stress: a systematic review. Sensors.

[2] (2017). Depression and Other Common Mental Disorders: Global Health Estimates. World Health Organization.

[3] Spijker J, De Graaf R, Bijl RV, Beekman ATF, Ormel J, Nolen WA (2002). Duration of major depressive episodes in the general population: Results from the Netherlands Mental Health Survey and Incidence Study (NEMESIS). Br J Psychiatry.

[4] Richards D (2011). Prevalence and clinical course of depression: A review. Clinical Psychology Review.

[5] Murray CJL, Lopez AD, World Health Organization, World Bank, Harvard School of Public Health (1996). The Global burden of disease: a comprehensive assessment of mortality and disability from diseases, injuries, and risk factors in 1990 and projected to 2020. World Health Organization.

[6] Eaton WW (1997). Natural history of Diagnostic Interview Schedule/ DSM-IV Major Depression. Arch Gen Psychiatry.

[7] Roca M, Armengol S, García-García M, Rodriguez-Bayón A, Ballesta I, Serrano MJ, Comas A, Gili M (2011). Clinical differences between first and recurrent episodes in depressive patients. Comprehensive Psychiatry.

[8] Tokgöz P, Hrynyschyn R, Hafner J, Schönfeld S, Dockweiler C (2021). Digital health interventions in prevention, relapse, and therapy of mild and moderate depression: scoping review. JMIR Ment Health.

[9] Zwerenz R, Baumgarten C, Becker J, Tibubos A, Siepmann M, Knickenberg RJ, Beutel ME (2019). Improving the course of depressive symptoms after inpatient psychotherapy using adjunct web-based self-help: follow-up results of a randomized controlled trial. J Med Internet Res.

[10] Maj M, Stein DJ, Parker G, Zimmerman M, Fava GA, De Hert M, Demyttenaere K, McIntyre RS, Widiger T, Wittchen H (2020). The clinical characterization of the adult patient with depression aimed at personalization of management. World Psychiatry.

[11] Cormack F, McCue M, Taptiklis N, Skirrow C, Glazer E, Panagopoulos E, van Schaik TA, Fehnert B, King J, Barnett JH (2019). Wearable technology for high-frequency cognitive and mood assessment in major depressive disorder: longitudinal observational study. JMIR Ment Health.

[12] Mehrotra A, Tsapeli F, Hendley R, Musolesi M (2017). MyTraces: investigating correlation and causation between users? Emotional states and mobile phone interaction. Proc ACM Interact Mob Wearable Ubiquitous Technol.

[13] Carney RM, Freedland KE (2009). Depression and heart rate variability in patients with coronary heart disease. CCJM.

[14] Burns MN, Begale M, Duffecy J, Gergle D, Karr CJ, Giangrande E, Mohr DC (2011). Harnessing context sensing to develop a mobile intervention for depression. J Med Internet Res.

[15] Rollo S, Antsygina O, Tremblay MS (2020). The whole day matters: Understanding 24-hour movement guideline adherence and relationships with health indicators across the lifespan. Journal of Sport and Health Science.

[16] Tazawa Y, Liang K, Yoshimura M, Kitazawa M, Kaise Y, Takamiya A, Kishi A, Horigome T, Mitsukura Y, Mimura M, Kishimoto T (2020). Evaluating depression with multimodal wristband-type wearable device: screening and assessing patient severity utilizing machine-learning. Heliyon.

[17] Moshe I, Terhorst Y, Philippi P, Domhardt M, Cuijpers P, Cristea I, Pulkki-Råback L, Baumeister H, Sander LB (2021). Digital interventions for the treatment of depression: A meta-analytic review. Psychological Bulletin.

[18] Lorenz N, Sander C, Ivanova G, Hegerl U (2020). Temporal associations of daily changes in sleep and depression core symptoms in patients suffering from major depressive disorder: idiographic time-series analysis. JMIR Ment Health.

[19] McDonald S, Quinn F, Vieira R, O’Brien N, White M, Johnston DW, Sniehotta FF (2017). The state of the art and future opportunities for using longitudinal n-of-1 methods in health behaviour research: a systematic literature overview. Health Psychology Review.

[20] Smith J Mobile eCommerce Stats in 2023: What Percentage of eCommerce Sales Are on Mobile Devices?. OuterBox Solutions.

[21] Bhalla N, Jolly P, Formisano N, Estrela P (2016). Introduction to biosensors. Essays Biochem.

[22] Moukaddam N, Truong A, Cao J, Shah A, Sabharwal A (2019). Findings from a trial of the Smartphone and OnLine Usage-based eValuation for Depression (SOLVD) application: what do apps really tell us about patients with depression? concordance between app-generated data and standard psychiatric questionnaires for depression and anxiety. J Psychiatr Pract.

[23] Jacobson NC, Weingarden H, Wilhelm S (2019). Using digital phenotyping to accurately detect depression severity. J Nerv Ment Dis.

[24] Gould CE, Carlson C, Ma F, Forman-Hoffman V, Ranta K, Kuhn E (2021). Effects of mobile app–based intervention for depression in middle-aged and older adults: mixed methods feasibility study. JMIR Form Res.

[25] Pedrelli P, Fedor S, Ghandeharioun A, Howe E, Ionescu DF, Bhathena D, Fisher LB, Cusin C, Nyer M, Yeung A, Sangermano L, Mischoulon D, Alpert JE, Picard RW (2020). Monitoring changes in depression severity using wearable and mobile sensors. Front Psychiatry.

[26] Marzano L, Bardill A, Fields B, Herd K, Veale D, Grey N, Moran P (2015). The application of mHealth to mental health: opportunities and challenges. The Lancet Psychiatry.

[27] Mohr DC, Zhang M, Schueller SM (2017). Personal sensing: understanding mental health using ubiquitous sensors and machine learning. Annu Rev Clin Psychol.

[28] Batra S, Baker R, Wang T, Forma F, DiBiasi F, Peters-Strickland T (2017). Digital health technology for use in patients with serious mental illness: a systematic review of the literature. MDER.

[29] Rohani DA, Faurholt-Jepsen M, Kessing LV, Bardram JE (2018). Correlations between objective behavioral features collected from mobile and wearable devices and depressive mood symptoms in patients with affective disorders: systematic review. JMIR Mhealth Uhealth.

[30] Cornet VP, Holden RJ (2018). Systematic review of smartphone-based passive sensing for health and wellbeing. Journal of Biomedical Informatics.

[31] Frank E, Pong J, Asher Y, Soares CN (2018). Smart phone technologies and ecological momentary data: is this the way forward on depression management and research?. Curr Opin Psychiatry.

[32] Köhnen M, Dreier M, Seeralan T, Kriston L, Härter M, Baumeister H, Liebherz S (2021). Evidence on technology-based psychological interventions in diagnosed depression: systematic review. JMIR Ment Health.

[33] Matcham F, Barattieri di San Pietro C, Bulgari V, de Girolamo G, Dobson R, Eriksson H, Folarin AA, Haro JM, Kerz M, Lamers F, Li Q, Manyakov NV, Mohr DC, Myin-Germeys I, Narayan V, BWJH P, Ranjan Y, Rashid Z, Rintala A, Siddi S, Simblett SK, Wykes T, Hotopf M (2019). Remote assessment of disease and relapse in major depressive disorder (RADAR-MDD): a multi-centre prospective cohort study protocol. BMC Psychiatry.

[34] Melbye S, Kessing LV, Bardram JE, Faurholt-Jepsen M (2020). Smartphone-based self-monitoring, treatment, and automatically generated data in children, adolescents, and young adults with psychiatric disorders: systematic review. JMIR Ment Health.

[35] Sequeira L, Perrotta S, LaGrassa J, Merikangas K, Kreindler D, Kundur D, Courtney D, Szatmari P, Battaglia M, Strauss J (2020). Mobile and wearable technology for monitoring depressive symptoms in children and adolescents: A scoping review. Journal of Affective Disorders.

[36] Wahle F, Bollhalder L, Kowatsch T, Fleisch E (2017). Toward the design of evidence-based mental health information systems for people with depression: a systematic literature review and meta-analysis. J Med Internet Res.

[37] Yim SJ, Lui LM, Lee Y, Rosenblat JD, Ragguett R, Park C, Subramaniapillai M, Cao B, Zhou A, Rong C, Lin K, Ho RC, Coles AS, Majeed A, Wong ER, Phan L, Nasri F, McIntyre RS (2020). The utility of smartphone-based, ecological momentary assessment for depressive symptoms. Journal of Affective Disorders.

[38] Liberati A, Altman DG, Tetzlaff J, Mulrow C, Gotzsche PC, Ioannidis JPA, Clarke M, Devereaux PJ, Kleijnen J, Moher D (2009). The PRISMA statement for reporting systematic reviews and meta-analyses of studies that evaluate healthcare interventions: explanation and elaboration. BMJ.

[39] Patil P, Sekar A Passive data collection: What it is and advantages. QuestionPro.

[40] Page MJ, McKenzie JE, Bossuyt PM, Boutron I, Hoffmann TC, Mulrow CD, Shamseer L, Tetzlaff JM, Akl EA, Brennan SE, Chou R, Glanville J, Grimshaw JM, Hróbjartsson A, Lalu MM, Li T, Loder EW, Mayo-Wilson E, McDonald S, McGuinness LA, Stewart LA, Thomas J, Tricco AC, Welch VA, Whiting P, Moher D (2021). The PRISMA 2020 statement: an updated guideline for reporting systematic reviews. BMJ.

[41] Long HA, French DP, Brooks JM (2020). Optimising the value of the critical appraisal skills programme (CASP) tool for quality appraisal in qualitative evidence synthesis. Research Methods in Medicine & Health Sciences.

[42] Saeb S, Lattie EG, Schueller SM, Kording KP, Mohr DC (2016). The relationship between mobile phone location sensor data and depressive symptom severity. PeerJ.

[43] Di Matteo D, Wang W, Fotinos K, Lokuge S, Yu J, Sternat T, Katzman MA, Rose J (2021). Smartphone-detected ambient speech and self-reported measures of anxiety and depression: exploratory observational study. JMIR Form Res.

[44] Di Matteo D, Fotinos K, Lokuge S, Mason G, Sternat T, Katzman MA, Rose J (2021). Automated screening for social anxiety, generalized anxiety, and depression from objective smartphone-collected data: cross-sectional study. J Med Internet Res.

[45] Di Matteo D, Fotinos K, Lokuge S, Yu J, Sternat T, Katzman MA, Rose J (2020). The relationship between smartphone-recorded environmental audio and symptomatology of anxiety and depression: exploratory study. JMIR Form Res.

[46] Rykov Y, Thach T, Bojic I, Christopoulos G, Car J (2021). Digital biomarkers for depression screening with wearable devices: cross-sectional study with machine learning modeling. JMIR Mhealth Uhealth.

[47] Teepe GW, Da Fonseca A, Kleim B, Jacobson NC, Salamanca Sanabria A, Tudor Car L, Fleisch E, Kowatsch T (2021). Just-in-time adaptive mechanisms of popular mobile apps for individuals with depression: systematic app search and literature review. J Med Internet Res.

[48] Boonstra TW, Nicholas J, Wong QJ, Shaw F, Townsend S, Christensen H (2018). Using mobile phone sensor technology for mental health research: integrated analysis to identify hidden challenges and potential solutions. J Med Internet Res.

[49] Jacobson NC, Summers B, Wilhelm S (2020). Digital biomarkers of social anxiety severity: digital phenotyping using passive smartphone sensors. J Med Internet Res.

[50] Deady M, Johnston DA, Glozier N, Milne D, Choi I, Mackinnon A, Mykletun A, Calvo RA, Gayed A, Bryant R, Christensen H, Harvey SB (2018). Smartphone application for preventing depression: study protocol for a workplace randomised controlled trial. BMJ Open.

[51] Pellegrini AM, Huang EJ, Staples PC, Hart KL, Lorme JM, Brown HE, Perlis RH, Onnela JJ (2022). Estimating longitudinal depressive symptoms from smartphone data in a transdiagnostic cohort. Brain and Behavior.

[52] Braund TA, Zin MT, Boonstra TW, Wong QJJ, Larsen ME, Christensen H, Tillman G, O’Dea B (2022). Smartphone sensor data for identifying and monitoring symptoms of mood disorders: a longitudinal observational study. JMIR Ment Health.

[53] Lee J, Solomonov N, Banerjee S, Alexopoulos GS, Sirey JA (2021). Use of passive sensing in psychotherapy studies in late life: a pilot example, opportunities and challenges. Front Psychiatry.

[54] Zulueta J, Piscitello A, Rasic M, Easter R, Babu P, Langenecker SA, McInnis M, Ajilore O, Nelson PC, Ryan K, Leow A (2018). Predicting mood disturbance severity with mobile phone keystroke metadata: a biaffect digital phenotyping study. J Med Internet Res.

[55] Jacobson NC, Chung YJ (2020). Passive sensing of prediction of moment-to-moment depressed mood among undergraduates with clinical levels of depression sample using smartphones. Sensors.

[56] Choudhary S, Thomas N, Ellenberger J, Srinivasan G, Cohen R (2022). A machine learning approach for detecting digital behavioral patterns of depression using nonintrusive smartphone data (complementary path to Patient Health Questionnaire-9 assessment): prospective observational study. JMIR Form Res.

[57] Everitt N, Broadbent J, Richardson B, Smyth JM, Heron K, Teague S, Fuller-Tyszkiewicz M (2021). Exploring the features of an app-based just-in-time intervention for depression. Journal of Affective Disorders.

[58] Zhou H, Al-Ali F, Kang GE, Hamad AI, Ibrahim RA, Talal TK, Najafi B (2020). Application of wearables to facilitate virtually supervised intradialytic exercise for reducing depression symptoms. Sensors.

[59] Opoku Asare K, Terhorst Y, Vega J, Peltonen E, Lagerspetz E, Ferreira D (2021). Predicting depression from smartphone behavioral markers using machine learning methods, hyperparameter optimization, and feature importance analysis: exploratory study. JMIR Mhealth Uhealth.

[60] Kathan A, Harrer M, Küster L, Triantafyllopoulos A, He X, Milling M, Gerczuk M, Yan T, Rajamani ST, Heber E, Grossmann I, Ebert DD, Schuller BW (2022). Personalised depression forecasting using mobile sensor data and ecological momentary assessment. Front Digit Health.

[61] Tønning ML, Faurholt-Jepsen M, Frost M, Bardram JE, Kessing LV (2021). Mood and activity measured using smartphones in unipolar depressive disorder. Front Psychiatry.

[62] McIntyre RS, Lee Y, Rong C, Rosenblat JD, Brietzke E, Pan Z, Park C, Subramaniapillai M, Ragguett R, Mansur RB, Lui LM, Nasri F, Gill H, Berriah S (2021). Ecological momentary assessment of depressive symptoms using the mind.me application: convergence with the Patient Health Questionnaire-9 (PHQ-9). Journal of Psychiatric Research.

[63] Sarda A, Munuswamy S, Sarda S, Subramanian V (2019). Using passive smartphone sensing for improved risk stratification of patients with depression and diabetes: cross-sectional observational study. JMIR Mhealth Uhealth.

[64] Fang Y, Forger DB, Frank E, Sen S, Goldstein C (2021). Day-to-day variability in sleep parameters and depression risk: a prospective cohort study of training physicians. NPJ Digit Med.

[65] Cho C, Lee T, Lee J, Seo JY, Jee H, Son S, An H, Kim L, Lee H (2020). Effectiveness of a smartphone app with a wearable activity tracker in preventing the recurrence of mood disorders: prospective case-control study. JMIR Ment Health.

[66] Mullick T, Radovic A, Shaaban S, Doryab A (2022). Predicting depression in adolescents using mobile and wearable sensors: multimodal machine learning–based exploratory study. JMIR Form Res.

[67] Zhang Y, Folarin AA, Sun S, Cummins N, Bendayan R, Ranjan Y, Rashid Z, Conde P, Stewart C, Laiou P, Matcham F, White KM, Lamers F, Siddi S, Simblett S, Myin-Germeys I, Rintala A, Wykes T, Haro JM, Penninx BW, Narayan VA, Hotopf M, Dobson RJ (2021). Relationship between major depression symptom severity and sleep collected using a wristband wearable device: multicenter longitudinal observational study. JMIR Mhealth Uhealth.

[68] Lee H, Cho C, Lee T, Jeong J, Yeom JW, Kim S, Jeon S, Seo JY, Moon E, Baek JH, Park DY, Kim SJ, Ha TH, Cha B, Kang H, Ahn Y, Lee Y, Lee J, Kim L (2022). Prediction of impending mood episode recurrence using real-time digital phenotypes in major depression and bipolar disorders in South Korea: a prospective nationwide cohort study. Psychol Med.

[69] Bai R, Xiao L, Guo Y, Zhu X, Li N, Wang Y, Chen Q, Feng L, Wang Y, Yu X, Wang C, Hu Y, Liu Z, Xie H, Wang G (2021). Tracking and monitoring mood stability of patients with major depressive disorder by machine learning models using passive digital data: prospective naturalistic multicenter study. JMIR Mhealth Uhealth.

[70] Colombo D, Palacios AG, Alvarez JF, Patané A, Semonella M, Cipresso P, Kwiatkowska M, Riva G, Botella C (2018). Current state and future directions of technology-based ecological momentary assessments and interventions for major depressive disorder: protocol for a systematic review. Syst Rev.

[71] Colombo D, Fernández-Álvarez J, Patané A, Semonella M, Kwiatkowska M, García-Palacios A, Cipresso P, Riva G, Botella C (2019). Current state and future directions of technology-based ecological momentary assessment and intervention for major depressive disorder: a systematic review. JCM.

[72] Firth J, Torous J, Nicholas J, Carney R, Pratap A, Rosenbaum S, Sarris J (2017). The efficacy of smartphone‐based mental health interventions for depressive symptoms: a meta‐analysis of randomized controlled trials. World Psychiatry.

[73] ICD-11: International Classification of Diseases 11th Revision. World Health Organization.

[74] (2013). Diagnostic and Statistical Manual of Mental Disorders, Fifth Edition.

[75] Kraus C, Kadriu B, Lanzenberger R, Zarate CA, Kasper S (2019). Prognosis and improved outcomes in major depression: a review. Transl Psychiatry.

[76] Wahle F, Kowatsch T, Fleisch E, Rufer M, Weidt S (2016). Mobile sensing and support for people with depression: a pilot trial in the wild. JMIR Mhealth Uhealth.

[77] Lisetti CL, Nasoz F (2004). Using noninvasive wearable computers to recognize human emotions from physiological signals. EURASIP J Adv Signal Process.

[78] Naughton F, Johnston D (2014). A starter kit for undertaking n-of-1 trials. The European Health Psychologist.

[79] Bietz MJ, Bloss CS, Calvert S, Godino JG, Gregory J, Claffey MP, Sheehan J, Patrick K (2016). Opportunities and challenges in the use of personal health data for health research. J Am Med Inform Assoc.

[80] De Angel V, Lewis S, White K, Oetzmann C, Leightley D, Oprea E, Lavelle G, Matcham F, Pace A, Mohr DC, Dobson R, Hotopf M (2022). Digital health tools for the passive monitoring of depression: a systematic review of methods. NPJ Digit Med.

[81] Adamakis M (2017). Comparing the validity of a GPS monitor and a smartphone application to measure physical activity. JournalMTM.

[82] Plasqui G, Bonomi AG, Westerterp KR (2013). Daily physical activity assessment with accelerometers: new insights and validation studies. Obesity Reviews.

[83] Schukat M, McCaldin D, Wang K, Schreier G, Lovell NH, Marschollek M, Redmond SJ (2018). Unintended consequences of wearable sensor use in healthcare. Yearb Med Inform.

[84] Canali S, Schiaffonati V, Aliverti A (2022). Challenges and recommendations for wearable devices in digital health: Data quality, interoperability, health equity, fairness. PLOS Digit Health.

[85] Doherty G, Coyle D, Sharry J (2012). Engagement with online mental health interventions: an exploratory clinical study of a treatment for depression. CHI '12: Proceedings of the SIGCHI Conference on Human Factors in Computing Systems.

[86] Riddell WC, Song X (2017). The role of education in technology use and adoption: evidence from the Canadian workplace and employee survey. ILR Review.

[87] Trull TJ, Ebner-Priemer UW (2020). Ambulatory assessment in psychopathology research: a review of recommended reporting guidelines and current practices. Journal of Abnormal Psychology.

